# Flutter sensitivity in FM bats. Part II: amplitude modulation

**DOI:** 10.1007/s00359-018-1292-y

**Published:** 2018-09-21

**Authors:** A. Leonie Baier, Kristin-Jasmin Stelzer, Lutz Wiegrebe

**Affiliations:** 1 Department Biology II, Ludwig Maximilians University Munich, Großhaderner Str. 2, 82152 Martinsried, Germany; 2 Acoustic and Functional Ecology Group, Max Planck Institute for Ornithology, Eberhard-Gwinner-Str. 11, 82319 Seewiesen, Germany

**Keywords:** Biosonar, Echolocation, SAM, Virtual target, Wagon-wheel effect

## Abstract

Bats use echolocation to detect targets such as insect prey. The echolocation call of frequency-modulating bats (FM bats) typically sweeps through a broad range of frequencies within a few milliseconds. The large bandwidth grants the bat high spatial acuity in depicting the target. However, the extremely short call duration and the overall low duty cycle of call emission impair the bat’s capability to detect e.g. target movement. Nonetheless, FM bats constitute more than 80% of all echolocating species and are able to navigate and forage in an environment full of moving targets. We used an auditory virtual reality approach to generate changes in echo amplitude reflective of fluttering insect wings independently from other confounding parameters. We show that the FM bat *Phyllostomus discolor* successfully detected these modulations in echo amplitude and that their performance increased with the rate of the modulation, mimicking faster insect wing-beats. The ability of FM bats to detect amplitude modulations of echoes suggests a release from the trade-off between spatial and temporal acuity and highlights the diversity of selective pressures working on the echolocation system of bats.

## Introduction

Bats emit ultrasonic calls and perceive targets such as insect prey by extracting information from the returning echoes. The short, broadband echolocation signals of frequency-modulating bats (FM bats) are very well suited to describe a three-dimensional static layout, due to their similarity to a Dirac Impulse (an impulse with infinitely broad bandwidth and infinitesimally short duration). As already argued in the first paper of this series (Baier and Wiegrebe this issue) though, short broadband calls emitted at low-duty-cycle (Fenton et al. 2012) are not well suited to describe a time-variant system, that is, movement of the ensonified target(s) and/or the bat itself. A frequency-modulated call (FM call) therefore grants high spatial acuity at the expense of accuracy in detecting the movement of the target. Nevertheless, more than 80% of echolocating bat species use FM calls (Nowak 1994).

The fluttering wings of edible insect prey generate a modulation of both echo delay and echo amplitude over time (Neuweiler 1984; Schnitzler et al. 1985). Therefore, bats’ ability to detect modulations of echo parameters is often referred to as flutter sensitivity. In our two-part study we used a virtual environment to investigate the two aspects of flutter sensitivity in bats: first, sensitivity to the modulation of echo delay and second, sensitivity to the modulation of echo amplitude.

In the first paper of the series (Baier and Wiegrebe this issue), we showed that despite the low-duty-cycle echolocation of the FM bat under study (*Phyllostomus discolor*) these bats can detect echo-delay modulations even as fast as 1000 Hz, which corresponds to the fastest wing beat rate found in insects (Sotavalta 1953). We demonstrated good sensitivity to modulations up to 10 Hz as well, but markedly worse sensitivity for an intermediate modulation range between 20 and 50 Hz. We discussed these results with respect to the different perceptual cues (nominal delay cues for slow modulations and Doppler distortions for fast modulations). We further proposed an echo-acoustic ‘wagon wheel effect’, i.e. an interaction of the modulation rate with the repetition rate of the bats’ emissions. Conspicuously the bats were worse at detecting echo-delay modulations when the modulation rate was in the same range as the call emission rate [within strobe groups of echolocation calls as they are typically emitted by our bats and many other FM bat species (Moss and Surlykke 2010)].

If the reason for the worse detection of intermediate delay-modulation rates lies in the wagon-wheel effect, one might predict that this would equally apply to the detection of the other echo feature that is periodically modulated by insect wing beats, namely the echo amplitude or target strength. Notably, echo delay and echo amplitude are encoded differently in the bat ascending auditory pathway (Hagemann et al. 2010; Hechavarría et al. 2013; Greiter and Firzlaff 2017; Measor et al. 2018). These considerations guided us in this second part of our two-part study where we explicitly address the question how sensitive the bat *P. discolor* is for modulations of echo amplitude over time.

To answer this question we used the auditory-virtual-reality setup that we already employed in the companion study (Baier and Wiegrebe this issue). The real-time digital signal processing allowed us to manipulate the modulation of amplitude at several modulation rates while leaving echo delay constant. We demonstrate that echo-acoustic sensitivity to echo-amplitude modulation did follow a different trajectory with modulation rate in comparison to sensitivity to echo-delay modulation. Further, we illustrate that at high modulation rates, spectral cues likely dominated the bats’ performance but that these spectral cues are not Doppler distortions.

## Materials and methods

For our two-part study we designed two behavioral experiments to manipulate first only the modulation of delay (Baier and Wiegrebe this issue) and second only the modulation of amplitude (current study). Here we used the same experimental animals and paradigm as in part one. For a detailed description of the methods, see therefore Baier and Wiegrebe, this issue.

### Animals and permit

We used four adult male individuals of the bat species *Phyllostomus discolor*, Wagner, 1843. All experiments were conducted under the regulations of the German Law on Animal Protection (approval 55.2-1-54-2532-34-2015, Regierung von Oberbayern).

### Virtual-target production

**Fig. 1:**
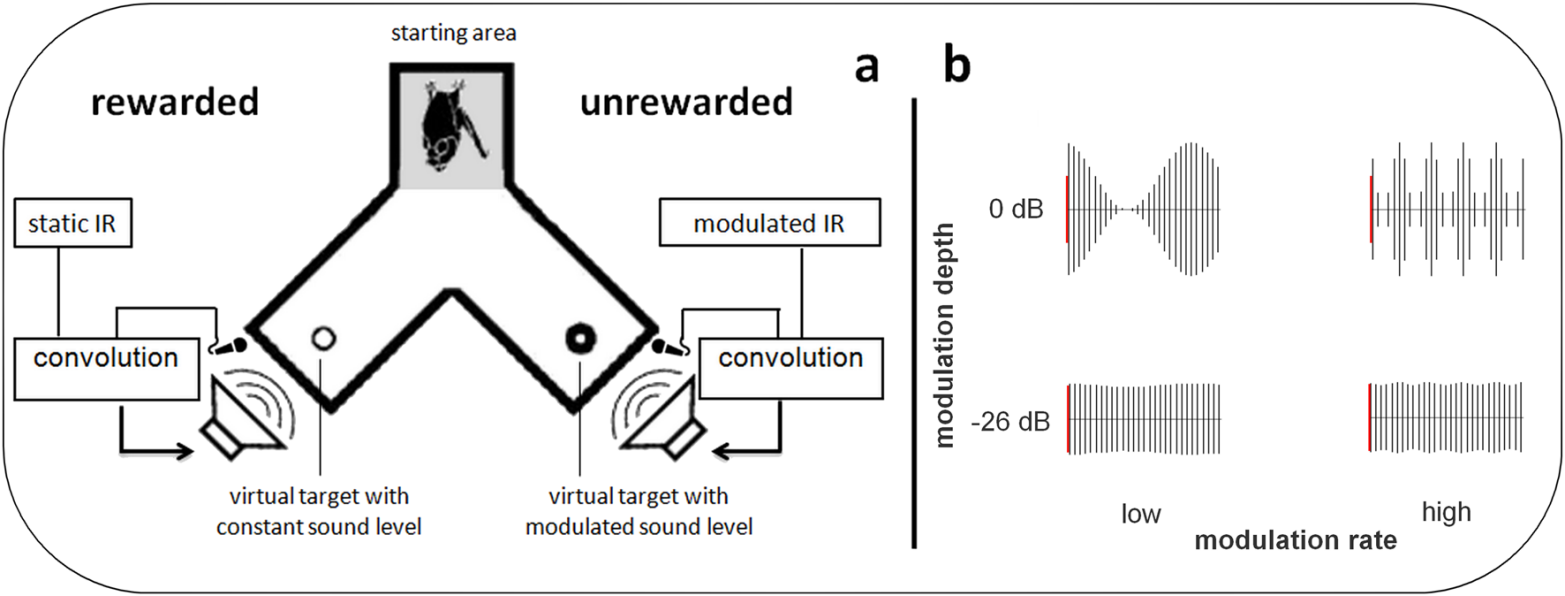
Auditory virtual reality setup. **a** Four bats were trained to discriminate a virtual target with constant sound level from a virtual target whose sound level was modulated at varying modulation rates ranging from 5 to 500 Hz. All bats learned to indicate the pseudorandomly chosen position of the static target by crawling toward it from the depicted starting area after echolocating toward both targets. Virtual targets were created by convolving recorded echolocation calls in real time with a static or time variant impulse response (IR). **b** The depth of the modulation ranged from 0 dB (corresponding to the sound level oscillating between silence and 200% of the input sound level) to − 26 dB (corresponding to the sound level oscillating between 95 and 105% of the input sound level). Red bars indicate 100% sound level. Black lines symbolize single echoes. Note that for visualization purposes call repetition rate is not to scale, but set about four times as high as we found in our bats

During the full length of a trial, a bat would utter echolocation calls. We recorded the emitted calls, implemented a time-variant amplitude modification and broadcasted the resulting virtual echoes via the loudspeakers, all in real-time (Fig. 1). Henceforth we will refer to the virtual echoes simply as echoes, although they were not echoes in the strict sense of an echo being a reflection from a physically present surface. Besides the modified echo amplitude, virtual targets were otherwise implemented as simple reflectors. Every change the bat chose to make in its emission sequence (e.g. change in call timing or call spectrum) was immediately reflected in the echoes. The only parameter that was systematically varied on our part was the echo amplitude (target strength).

We used two impulse responses representing two virtual targets: The target strength of one target was kept constant while the target strength of the other target was modulated over time, i.e., the amplitude of one echo was constant while the other was modulated. We tested the sensitivity of the bats to amplitude modulation at six different modulation rates: 5, 10, 20, 50, 200, and 500 Hz. At a modulation rate of 5 Hz, the signal undergoes one full modulation period within 200 ms, i.e., from original loudness to amplification, to original loudness, to attenuation, and finally to original loudness again. We tested modulation depths decreasing in steps of 2 dB, starting from 0 dB until a particular bat’s threshold was found (see “Behavioral procedure” section). The modulation depth of 0 dB corresponds to 100% modulation (easiest condition), i.e. the amplitude oscillates between twice the linear amplitude of the unmodulated target and silence. The modulation depth of − 26 dB corresponds to 5% modulation, i.e. the linear amplitude oscillates only between 105% and 95% of the linear amplitude of the unmodulated target (most difficult condition; Fig. 1b).

### Behavioral procedure

We quantified sensitivity for the modulation of echo amplitude with a formal psychophysical experiment, following a two-alternative, forced-choice (2AFC) paradigm. Bats were offered a choice between the constant and the modulated target and were trained to choose the constant target for a food reward. A bat indicated its choice by crawling down one arm of a *y*-shaped maze towards the target and the corresponding feeder (Fig. 1a). For each bat we recorded a full psychometric function per modulation rate. A psychometric function describes the relationship between the stimulus magnitude and the subject’s response. Here we looked at the relationship between the depth of the modulation and the bat’s performance on the discrimination of constant vs. modulated target. Discrimination performance was measured in percent correct choices over a minimum of 30 trials per modulation depth. We tested modulation depths ranging from 0 to − 26 dB in steps of 2 dB. For each bat, data collection was complete on reaching a modulation depth below threshold, i.e., where performance dropped below 70% for two consecutive modulation depths. Each completed psychometric function consisted of performance measures at at least seven different modulation depths (≥ 210 trials). The threshold extracted from the psychometric function is the just-noticeable difference (JND) in modulation for the respective bat at the respective modulation rate. We fitted the psychometric function with a sigmoidal function and estimated the threshold for the respective animal and modulation rate from the fit’s value at 70% correct performance (*p* < 0.05, binomial test). The entirety of the extracted threshold values described the sensitivity of *P. discolor* for the modulation of echo amplitude across a wide range of modulation rates.

### Acoustic analyses

The echo properties depended both on the properties of the virtual targets themselves and critically on the properties of the emitted calls that the bats used to ensonify them. In our study, we manipulated the echo-acoustic target properties. We verified the echolocation-call properties with sound analysis. We simulated the resulting echo properties with regard to the nominal perceptive cue (amplitude modulation) and to additional perceptive cues that may serve the bats to detect the echo-amplitude modulations.

For sound analysis, the recorded call sequences were saved parallel to the virtual-target production. After high-pass filtering the stereo recordings at 35 kHz (8th order butterworth filter), we extracted echolocation calls and calculated temporal and spectral call parameters from the channel with higher call level (inter-call interval and-10 dB call duration; spectral centroid, i.e., weighted mean of the frequencies present in the signal, based on time-averaged spectrogram with a 750 Hz binwidth). Due to technical problems we were forced to replace the original audio-interface (Motu Ultralite, Motu, Cambridge, MA, USA) used to record echolocation calls during ongoing data acquisition. The new interface’s (RME Fireface 400, Audio AG, Haimhausen, Germany) recording properties introduced a downward shift in the frequency content of recorded calls compared to older recordings.

For the simulation of perceived echo cues we recreated the virtual target’s time-variant impulse response (i.e. the modulator) as a sine wave and multiplied it with either recorded echolocation-call sequences or artificial echolocation calls (multiharmonic FM-downward sweep of 1 ms duration with fundamental frequency ranging from 23 to 19 kHz). First, we looked at the nominal cue, i.e. at the amplitude differences between two consecutive echolocation calls. Due to the time-variant nature of the impulse response, there were no two fixed echo amplitudes that constituted the difference, but a range of potential amplitudes, depending on both the bats’ timing of echolocation call emission and the phase at which the emitted calls hit the modulator. We used eight different modulator phases to simulate a comprehensive subset of possible call-to-call amplitude differences. The amplitude difference between two consecutive echoes naturally also depends on the inter-call interval (ICI). For our simulations we used real ICIs extracted during sound analysis. Second, we addressed potential additional cues that might arise from the interaction of one single echolocation call with the modulator. Again, the outcome depends on the modulator phase that the call interacts with. We thus simulated a subset of possible interactions by multiplying one artificial call with eight different modulator phases.

## Results

### Behavioral response

Four male FM bats (*Phyllostomus discolor*) learned to discriminate between a virtual echo with constant amplitude and a virtual echo with modulated amplitude. We used the behavioral response of the bats to assess the just noticeable modulation depth, i.e. the threshold. For every bat we extracted one threshold per modulation rate from the psychometric function to form a modulation transfer function across the six modulation rates. It describes the sensitivity of the FM bat *Phyllostomus discolor* for the modulation of echo amplitude.

**Fig. 2:**
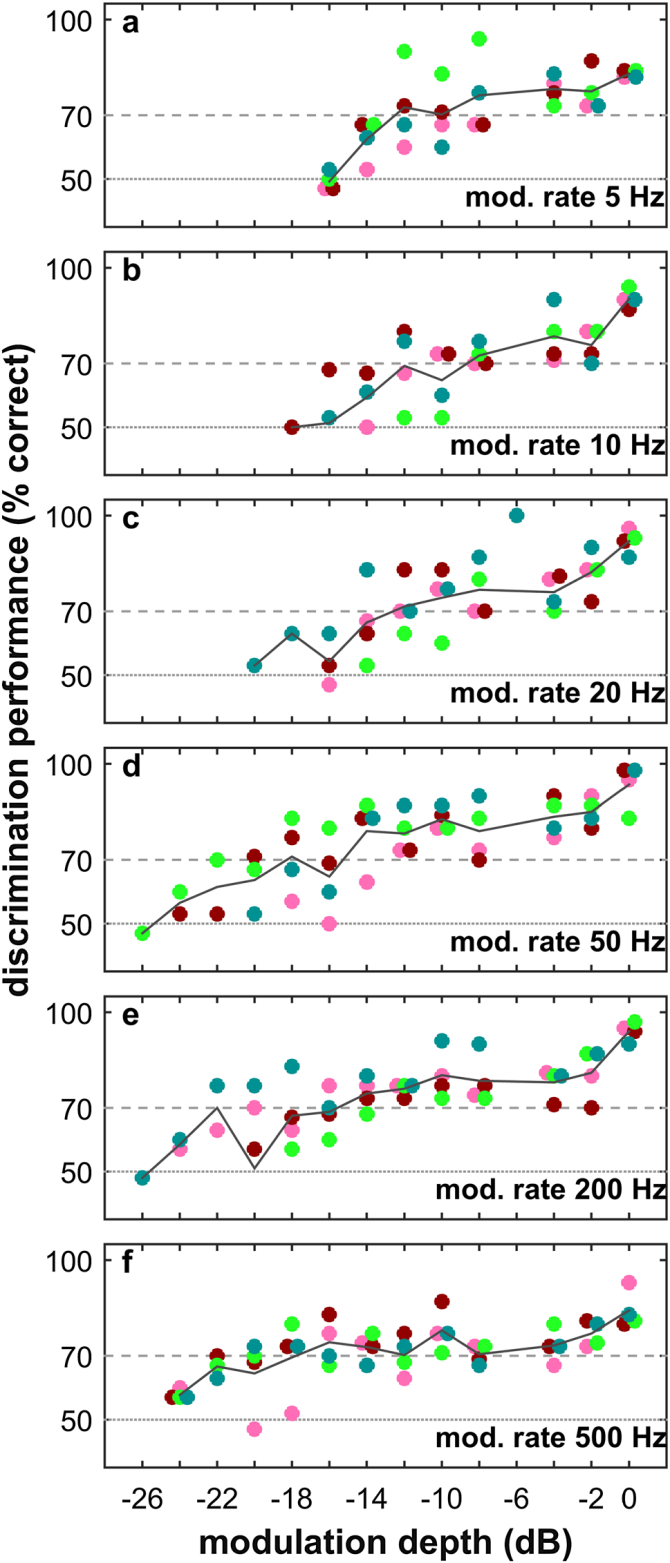
Psychometric functions of echo-amplitude-modulation discrimination performance at six modulation rates. Each colored dot marks one bat’s discrimination performance across 30 trials. Black line plots depict the discrimination performance averaged across bats. Horizontal dashed lines at 50 and 70% correct depict chance and significance level, respectively

In the discrimination of constant vs. modulated target, the results of all four bats confirmed our expectations for a psychometric function: The discrimination performance of all bats was best at the largest modulation depth of 0 dB. All bats discriminated the constant target from the amplitude-modulated target at 80%–100% correct choices level. With decreasing modulation depth, the performance deteriorated and eventually dropped to chance level. We observed this relationship for all modulation rates (Fig. 2a–f).

However, the extent of the psychometric function systematically widened towards smaller modulation depths with increasing modulation rate. Starting from the lowest modulation rate of 5 Hz (Fig. 2a), where the average discrimination threshold lay between − 10 and − 12 dB amplitude modulation, the bats’ average performance improved with increasing modulation rate. For example, at a modulation rate of 50 Hz the average discrimination threshold lay between − 16 and − 18 dB amplitude modulation (Fig. 2d). At the highest tested modulation rate of 500 Hz, the average discrimination threshold lay between − 18 and − 20 dB amplitude modulation (Fig. 2f).

**Fig. 3:**
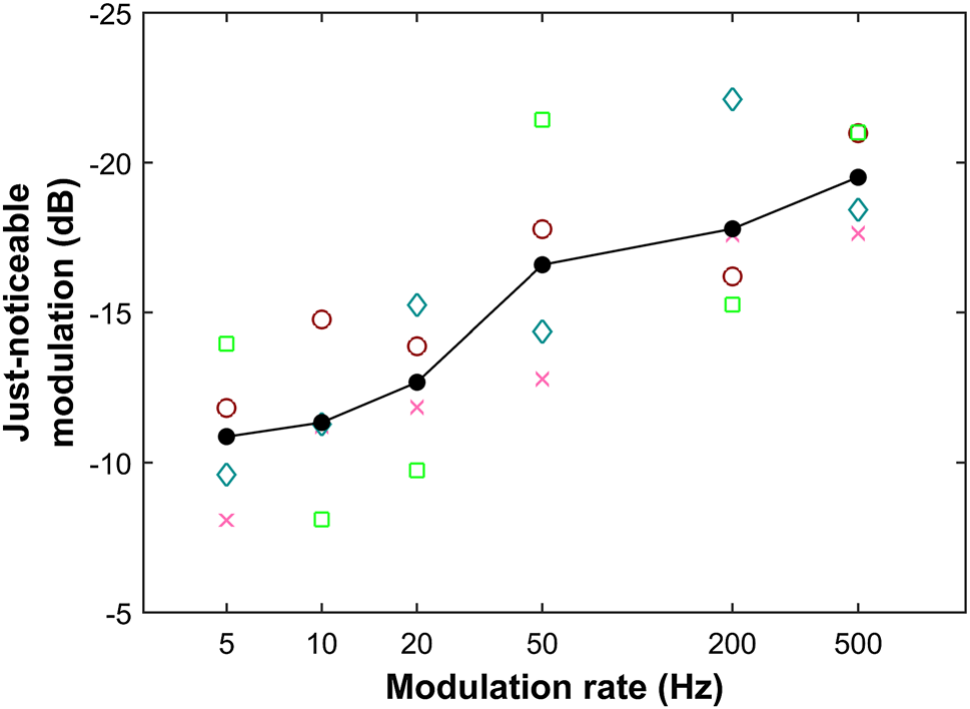
Echo-amplitude modulation sensitivity. Detection thresholds are generally worst at the lowest modulation rate and improve with increasing modulation rate. Marker colors represent individual bats’ thresholds as extracted from sigmoidal fits to the psychometric functions in Fig. 2, the black line connects the mean values of all four bats

The just-noticeable modulation values extracted from the six psychometric functions form the modulation transfer function that describes the bats’ sensitivity for echo-amplitude modulation across six modulation rates (Fig. 3). The modulation transfer function shows that overall sensitivity increases with increasing modulation rate: bats performed best at the highest modulation rate of 500 Hz with thresholds around − 20 dB modulation depth. For the lowest modulation rate of 5 Hz, the bats needed around − 10 dB modulation depth to discriminate the constant target from the modulated one.

### Acoustic analyses

The bats’ auditory percept depended not only on the echo-acoustic features of the virtual targets themselves but critically on how the bats ensonified them. We performed acoustic analyses of the echolocation calls used by the bats during the behavioral experiment to better understand which sensory- and vocal-motor strategies the bats employed to solve the task. Additionally, we simulated echo properties with respect to the nominal perceptive cue (echo-amplitude modulation) and to possible other perceptive cues.

**Fig. 4:**
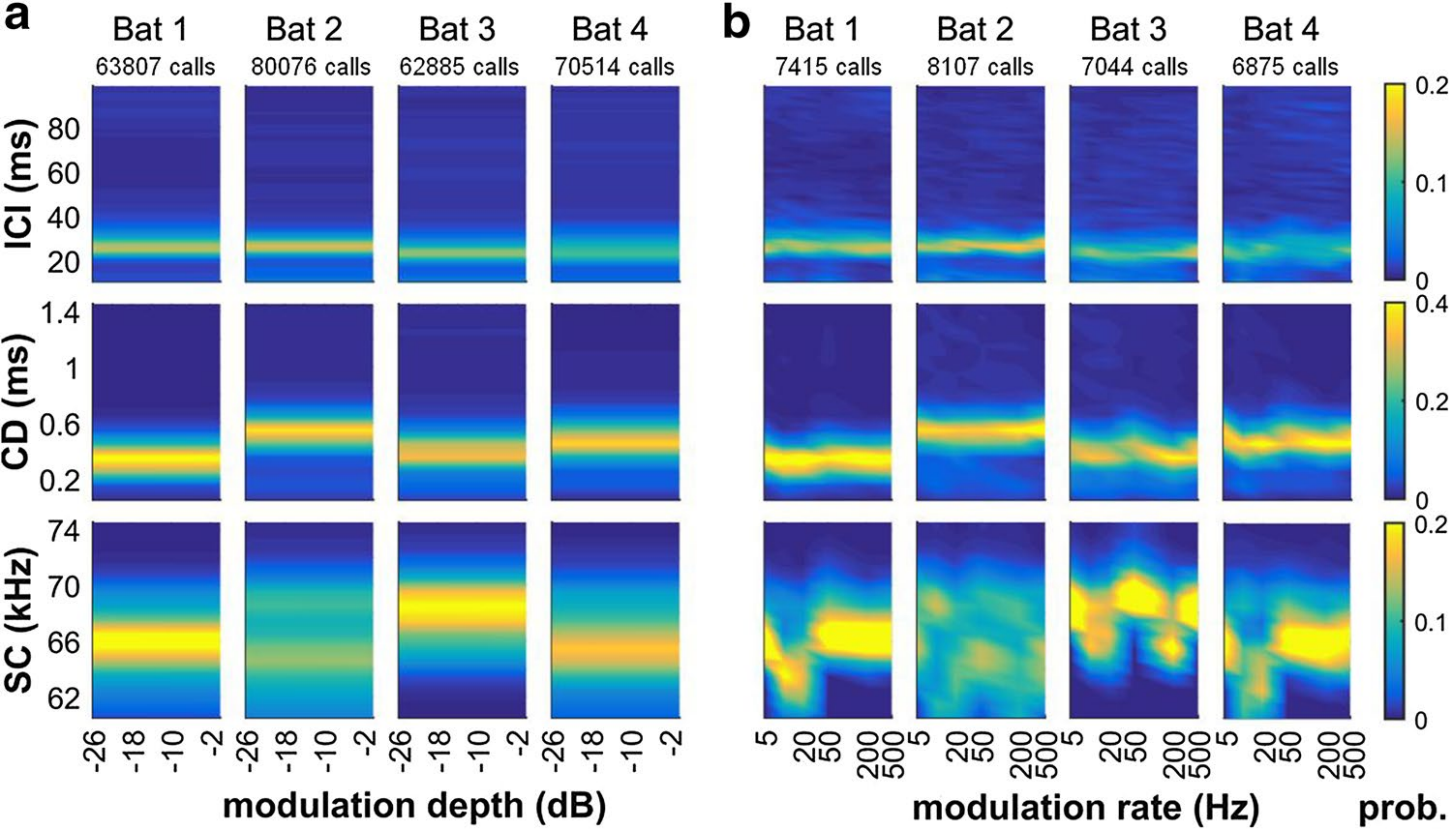
Temporal and spectral properties of echolocation calls used by the bats for detecting echo amplitude modulations. **a** Effect of modulation depth (= task difficulty). The distribution of inter-call intervals (Row 1), call durations (Row 2) and spectral centroids (Row 3) did not change as a function of modulation depth in any of the four bats (columns). **b** Effect of modulation rate at threshold modulation depth. Data show the extent to which the presented modulation rate lead the animals to adjust ensonification parameters. Again, the distribution of inter-call intervals (Row 1), call durations (Row 2) and spectral centroids (Row 3) did not change systematically as a function of presented modulation rate in the four bats. Residual changes in spectral-centroid distributions result from a change of recording device during data acquisition and do not reflect a change in the bats’ ensonification strategy. All data are shown as normalized bin counts with color-coded probability

In the acoustic analysis of the echolocation calls we first determined whether fundamental call parameters like inter-call intervals (ICIs), call duration, or the spectral centroid of the calls changed systematically when the task became more difficult for the bats, i.e., when the modulation depth decreased. We found that the bats did not systematically modify any of these call parameters with increasing task difficulty (Fig. 4a). Second we tested whether these call parameters changed systematically with modulation rate. Here we used only data from those trials where modulation depth was close to the perceptual threshold for this modulation rate and bat. Again, temporal ensonification parameters of the bats remained rather constant as a function of modulation rate, albeit different across individual bats (Fig. 4b, Rows 1 and 2). The changes in spectral centroid across modulation rates (Fig. 4b, Row 3) do not reflect a change in the bats’ ensonification strategy but a change in our recording setup that became necessary during ongoing data acquisition (see “Materials and methods” section). Affected were trials testing modulation rates of 200 Hz, 20 Hz and 10 Hz, depending on the individual bat’s progress at the point of the hardware change.

**Fig. 5:**
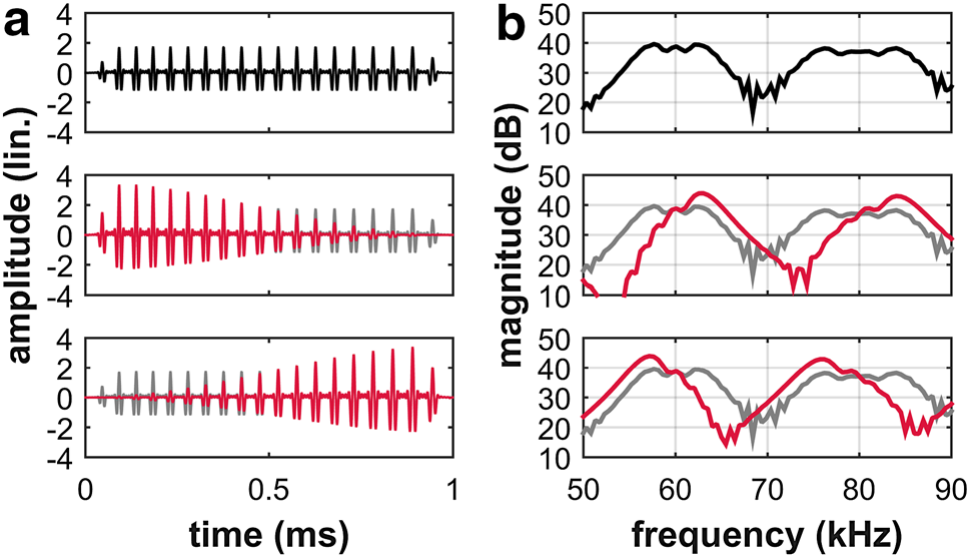
Simulated time signals (**a**) and zoomed-in power spectra (**b**) of echoes from an unmodulated reflector (top row), from a reflector with falling amplitude (middle row) and from reflector with rising amplitude (bottom row). **a** When the modulation rate is high enough, i.e. the modulation period is about the length of the echo, the echo amplitude changes significantly during the duration of one echo. **b** Due to the downward frequency modulation of the call, i.e. high frequencies are followed by intermediate and then low frequencies within the call’s duration, different frequency bands get emphasized and suppressed in modulated echoes (red) in comparison to the unmodulated echo (black/gray)

For the simulation of echo properties, we used a sequence of artificial echolocation calls and analyzed amplitude differences between consecutive echoes within the resulting sequence of artificial echoes. The bats’ sensitivity for echo-amplitude modulation increased with increasing modulation rate. Therefore the modulation of echo amplitude at threshold decreases with increasing modulation frequency: for modulation rates up to 20 Hz, the maximum amplitude differences between consecutive echoes were around ± 5 dB. For modulation rates of 50 Hz and above, the maximum amplitude differences at threshold decreased to ± 3 dB at 500 Hz (data not shown). *P. discolor* can discriminate echoes from two separate targets that differ in amplitude by 5–7 dB (Heinrich et al. 2011). When at high modulation rates the amplitude differences become smaller, other echo parameters besides the variation of echo amplitude may facilitate the psychophysical task: specifically, bats can analyze variations in the spectral composition of echoes (Schmidt 1988; Weissenbacher and Wiegrebe 2003; Falk et al. 2011). In another simulation, we tested whether fast amplitude modulations can induce perceivable changes in echo spectral composition. We analyzed the frequency content of the echoes and found that depending on the phase of the modulation relative to the timing of the call emission, the target amplitude modulation can indeed affect the echo spectrum (Fig. 5). The reason is that the time-variant amplitude of the virtual target interacts with the time-variant frequency content of the echolocation call: when at echo onset the virtual-target amplitude is high, the high frequencies of the echoes are stressed. When, however, at echo onset the target amplitude is low and increasing during the echo, the low frequencies of the echo are stressed. These target-amplitude induced variations in echo spectral content can only come about when the modulation period (1/modulation rate) is in the range of the echo duration. Moreover, the changes in echo spectrum depended on the phase of the modulation, so that consecutive echoes were subjected to different frequency changes.

## Discussion

When a bat echolocates towards a real target, the delay and amplitude of the echo change together, dependent on the distance of the target. Here we show that FM bats are very sensitive to modulations in amplitude of echoes: *Phyllostomus discolor* bats were well able to distinguish a virtual target with constant target strength from a virtual target whose target strength was modulated over time. To the best of our knowledge, this study presents the first evidence of an echolocating FM bat detecting a target movement based solely on the modulation of echo-amplitude.

### Amplitude-modulation sensitivity

The capability of FM bats to perceive amplitude modulations in echo sequences does not come as a complete surprise (Griffin 1958; Roeder 1963). But our results are the first to experimentally support the implications of theoretical studies on perception of motion with FM echolocation. In those studies, fluttering insects were ensonified with a sequence of synthetic FM signals, and information on wing beat cycle could be decoded from the resulting sequence of echoes (Kober and Schnitzler 1990; Moss and Zagaeski 1994).

The neural adaptations to process echo-amplitude information have been established for the FM bat *Myotis lucifugus* (Condon et al. 1994): neurons in the inferior colliculus faithfully represent amplitude modulations imposed on a sequence of artificial echolocation calls up to modulation rates around 100 Hz. However, there are three fundamental differences between the electrophysiological experiment by Condon et al. (1994) and the current psychophysical experiment. First, we varied modulation depth to determine a perceptual threshold while Condon et al. always applied 100% (0 dB) modulation depth. Second, the carrier was always a periodic pulse train, whereas in our experiments, the bats themselves determined the ensonification pattern, producing pulses in strobe groups rather than periodically. Third, the combination of pulse rate and modulation rate was always chosen to deliberately avoid a wagon wheel effect in the physiological studies. In summary, this electrophysiological study investigated the effect of amplitude modulation on the neural representation of echo sequences, but the limitations of the electrophysiological protocol preclude a direct comparison with the current psychophysical data.

*P. discolor* bats were much more sensitive to higher modulation rates than to lower modulation rates: for the lowest modulation rate of 5 Hz, the bats needed around − 10 dB modulation depth to discriminate the constant target from the modulated one, whereas they still detected modulations around − 20 dB modulation depth at the highest modulation rate of 500 Hz (Fig. 3). Strikingly, our findings that sensitivity increased with increasing modulation rate are inconsistent with two related findings: First, they differ from what we found about sensitivity to modulation of echo delay (Baier and Wiegrebe this issue). This suggests that time-variant information on delay and on amplitude of echoes is processed differently. Second, the results are the opposite of what has been found in studies where subjects passively listened to amplitude-modulated sounds (Fay and Wilber 1989). With broadband carrier stimuli, modulation transfer functions in these experiments are always low-pass, i.e., subjects are less sensitive to high modulation rates than to low modulation rates. This is in direct contrast to the results of the current, active echolocation study. We will discuss both points in detail after addressing the bats’ acoustic signals.

### Acoustic analyses

As expected from the delay study (Baier and Wiegrebe this issue), individual bats maintained the same call parameters throughout the experiment (Fig. 4). In fact, call parameters were identical to the ones used by the bats in the delay study: we found call durations around 0.4 ms and inter-call intervals (ICIs) around 29 ms. We propose that call parameters were tied to the here simulated target distance of 72 cm, although the bats were not in a formal target-approach situation, where a systematic decrease in ICI and call duration occurs (Griffin et al. 1960). We can ask whether changing the bat’s perceived target distance will result in different call parameters and lead to a different performance in the detection task. Echo sound pressure level (SPL) influences the performance in a range detection task (Denzinger and Schnitzler 1994, 1998). The sensitivity of neurons to sinusoidal amplitude modulation (SAM) is linked to the sound level of the carrier tone (Schuller 1979; Ostwald et al. 1988). Since bats could move freely in our experimental setup, we did not attempt to measure echo SPL. However, we had designed our virtual targets as plain reflectors that very likely yielded target strengths large enough to not affect the modulation detection. The question whether our chosen target range did so remains open.

### Comparison to echo-delay modulation

In the companion paper, we have investigated sensitivity for the modulation of echo delay (Baier and Wiegrebe this issue). We found that *P. discolor* bats were very sensitive to low and high delay-modulation rates and much less so to intermediate delay-modulation rates around 20 and 50 Hz. We proposed that for delay-modulation rates of 20 Hz and higher, bats suffer from an auditory wagon-wheel effect because their call repetition rate matches the modulation rate of the target or an integer multiple thereof. In these cases, the difference in echo delay between consecutive echoes becomes undetectably small. The use of spectral cues that only occur at high modulation rates and are not affected by this wagon-wheel effect can explain the recovery of detection performance for modulation rates of 100 Hz and higher. If we assume that for the detection of echo-amplitude modulation bats use amplitude differences between consecutive echoes, we should observe a similar wagon-wheel effect for the current sensitivity for amplitude modulation. However, we did not observe a drop in performance at intermediate amplitude-modulation rates (Fig. 3). This indicates that the currently observed increasing sensitivity for increasing amplitude-modulation rates does not purely reflect the ability to detect echo-amplitude differences. It rather indicates that the bats employ a different detection strategy already at low modulation rates where the wagon-wheel effect does not yet occur.

**Fig. 6:**
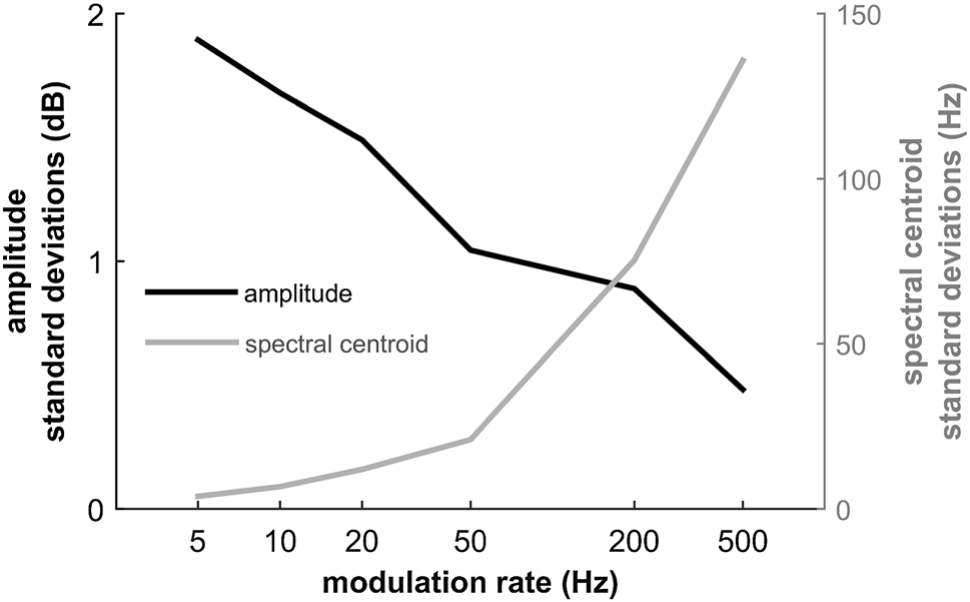
Standard deviations for amplitude and for spectral centroid of echoes from an artificial echolocation call at eight different modulator phases. The amount of variation in the amplitude of modulated echoes decreases with modulation rate (black line). In contrast, the amount of variation in the spectral centroid of modulated echoes increases with modulation rate (gray line). The modulation depth of the modulator for each modulation rate was set to the respective average detection threshold (black markers in Fig. 3)

We propose a detection strategy that depends on spectral cues rather than amplitude cues. We simulated the interaction of a frequency-modulated echolocation call with a fast amplitude modulation and observed that in an amplitude-modulated echo, different frequency bands get emphasized and suppressed in comparison to an unmodulated echo (Fig. 5). We quantified these changes by calculating the weighted mean of the frequencies present in the echo, i.e., the echo spectral centroid. The variation in echo amplitude between different phases of the amplitude modulation decreased with increasing modulation rates (Fig. 6, black line). In direct contrast to this, variation in spectral centroid between different phases of the amplitude modulation increased with increasing modulation rates (Fig. 6, gray line). In other words, amplitude cues become less and spectral cues become more available with increasing modulation rate. The bats’ behavioral performance in detecting amplitude modulations can therefore be explained by a shift in the emphasis that they place on processing different auditory cues. We can only guess at the details of the perceptual weighting that the bats might apply towards amplitude and spectral (and possibly further) cues, however, the high-pass shape of the behavioral modulation transfer function suggests a strong contribution of the spectral cues.

**Fig. 7:**
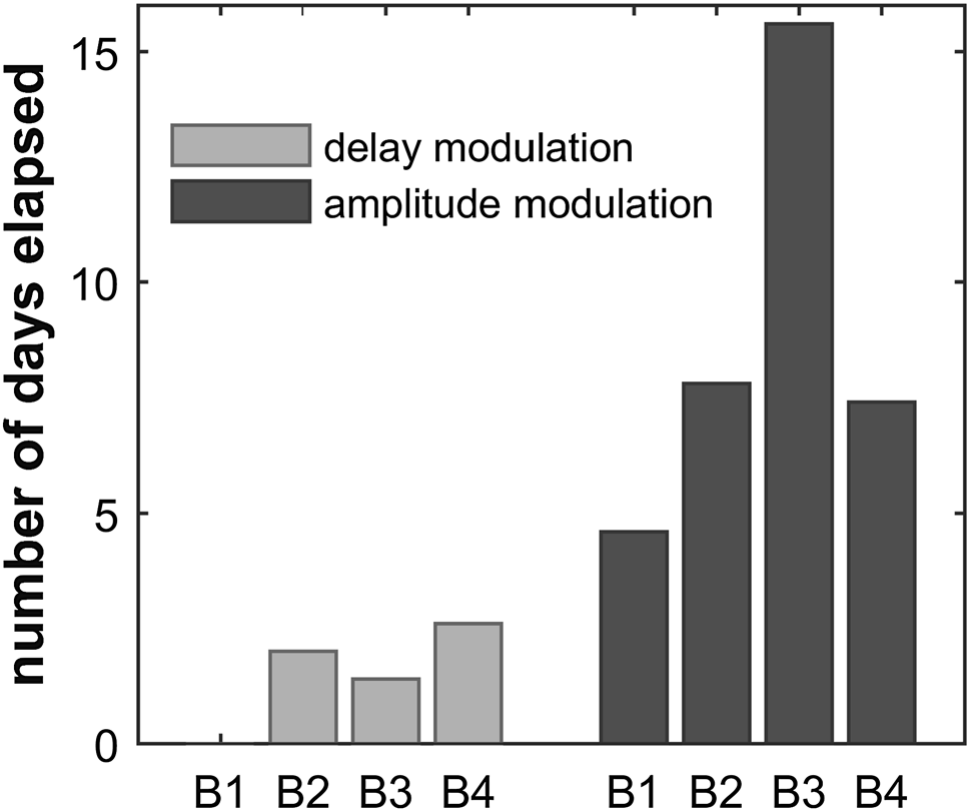
Average time between start of training and start of data collection for delay-modulation and amplitude-modulation experiments. During the echo-delay modulation experiment, it took the bats on average 1.5 days to learn a new modulation rate (light bars, *n* = 4). During the echo-amplitude modulation experiment, the same individual bats needed on average 8.9 days to learn a new modulation rate (dark bars, *n* = 4). B1–B4 refers to bats, the same individuals in both experiments

The idea that the perception of echo-amplitude modulation is processed differently from the perception of echo-delay modulation is corroborated by observations during the training procedure: all four bats that participated in the current amplitude-modulation experiments had previously participated in the delay-modulation experiments. The rewarded stimulus in these two experiments was the same unmodulated reflection. The bats were thus familiar with virtual targets, the experimental set-up and procedure. However, while these bats needed very little retraining when we wanted to acquire data for a new delay modulation rate, the same individuals needed extensive retraining periods when we wanted to acquire data for a new amplitude modulation rate. Retraining periods are illustrated in Fig. 7. The data clearly show that on average the animals needed more than five times the retraining time when we changed the amplitude-modulation rate than when we changed the delay-modulation rate. These data, together with the difference in psychophysical performances suggest that the modulation of echo amplitude may be processed fundamentally differently from the modulation of echo delay. We hypothesize that the dedicated neural circuitry for echo delay, and specifically the topographic representation of echo delay in the bat auditory cortex, supports delay-modulation detection (Hagemann et al. 2010; O’Neill and Suga 1979; Dear et al. 1993), whereas the lack of a similar topographic representation for echo amplitude may underlie the much more demanding training and data acquisition for amplitude-modulation detection. Modulation at high rates induces spectral cues for both echo-delay modulation and echo-amplitude modulation. These spectral cues are readily represented along the tonotopic axes at virtually all stages of the bat auditory system. The fact that our bats learned to detect fast delay modulations much quicker than fast amplitude modulations may be related to the fact that the delay-induced Doppler distortions create overall much more dramatic spectral distortions than the above described spectral changes induced by the echo-amplitude modulation.

### Comparison to passive listening

In passive listening, studies that investigate sensitivity to amplitude modulation typically use broadband noise as the carrier signal for the modulation, as opposed to a sequence of echoes of self-produced, broadband echolocation calls. The sensitivity of mammals and birds to amplitude modulation of noise consistently becomes worse with increasing modulation rate (Viemeister 1979; Salvi et al. 1982; Burdin et al. 1973; Dooling and Searcy 1981).

These findings are in direct contrast to the results we present here for the sensitivity of bats to amplitude modulation of echoes, and indicate separate processing mechanisms for amplitude modulations in passive and active listening, at least for part of the range of modulation rates. This discrepancy can be assigned to the fundamental difference in the nature of the modulated sounds. In passive listening, the carrier of the amplitude modulation is a continuous signal that persistently reflects every point of the modulation. In active listening, the carrier of the amplitude modulation is a transient, i.e. impermanent signal that reflects only momentary stages of the modulation.

When the carrier of the amplitude modulation in passive listening is a pure tone, there also arise spectral cues that allow the detection sensitivity of human listeners to recover for modulation rates higher than 50 Hz (Viemeister 1979). Fast amplitude modulation of a narrowband signal creates spectrally resolvable sidebands: when a 1000 Hz pure tone is amplitude-modulated at a modulation rate of 200 Hz, the sidebands are at 800 and 1200 Hz. If we regarded the frequency-modulated broadband echolocation call as an assembly of pure-tone carriers, the broadband echo itself would mask any emerging frequency sidebands, even at the highest presented amplitude modulation rate of 500 Hz. Instead, the observed changes in the frequency spectrum of amplitude-modulated echoes (Figs. 5, 6) are likely to serve the same purpose as frequency sidebands in passive listening, complementing the detection of echo-amplitude modulation for those (high) modulation rates where this strategy is more efficient than resolving temporal amplitude differences.

Dankiewicz et al. (2002) performed the only other perceptual study on amplitude modulation detection with echolocation, i.e. not in passive listening. They trained a bottlenose dolphin (*Tursiops truncatus*) to discriminate modulated synthetic echoes from unmodulated ones. In contrast to the current work, the authors did not generate real-time echoes from the animal’s emissions but only used the animal’s emission to trigger the playback of the synthesized echo. Second, the authors applied one amplitude modulation cycle across a fixed number of echoes (8 to 64), not across a fixed time; thus the effective modulation frequency depended on the number of the dolphin’s emissions per time. Modulation detection thresholds changed from about 1.2% (= − 38 dB) for an effective modulation frequency around 2 Hz to a threshold of about 6% (= − 24 dB) for an effective modulation frequency around 16 Hz. Thus the modulation transfer function was low-pass, similar to what has been reported for amplitude-modulation detection with wideband noise carriers in humans (Viemeister 1979). Although the authors did not test the dolphin at effective modulation rates higher than 16 Hz, the general potential for spectral echo changes is low: interactions between the amplitude modulation of the virtual target and the frequency modulation of the emitted call are limited in the dolphin, because its average echo duration is markedly shorter than in our bats [128 µs and 500 µs, respectively (Dankiewicz et al. 2002)]. Instead, with regard to perceiving amplitude differences between consecutive echoes at very small modulation depths, echolocating dolphins are presumably superior to bats, as they have repeatedly been reported to be capable of detecting a 1 dB difference in target strength (Evans 1973; Bullock et al. 1968; Johnson 1967; Moore et al. 1995) compared to 5–7 dB in *P. discolor* (Heinrich et al. 2011). Additionally, dolphins may have no need to detect modulation rates that would be high enough to create spectral cues. While echo-amplitude modulation can reflect the changing orientation of the target relative to the emitter both in bats and dolphins, the speed of these orientation changes is likely much slower underwater than in air. All of these aspects make dolphins more likely to solely use echo-amplitude cues, resulting in their reported low-pass modulation transfer function.

As opposed to dolphins, FM bats would greatly benefit from a mechanism to facilitate flutter detection at high modulation rates, because these make up a large portion of insect prey wing beat rates (Pringle 1949; Sotavalta 1953; Gibson et al. 2010). Bats from the Eocene radiation were nocturnal and insectivore (Speakman 2001; Veselka et al. 2010). The surprisingly good performance of our bats (especially at higher modulation rates) leads us to hypothesize that the biophysical properties of FM echolocation calls do indeed facilitate insect flutter detection, not only based on echo-delay modulations, but also based on echo-amplitude modulations. We suggest that the frequency-modulated call structure does not only reflect a motor constraint of laryngeal echolocation or a means to minimize Doppler-distortion-induced misjudgments in echo delay (Altes 1995; Simmons et al. 2004). We argue that adaptive selection to insect prey motion shaped FM echolocation calls not into a perfect Dirac Impulse but into a structure that converts fast amplitude modulations into spectral cues, which are readily represented along the bats’ tonotopic axis. We surmise that the small deviation from the Dirac structure grants FM bats sensitivity to fast-changing time-variant environments together with high spatial acuity in time-invariant scenarios.

In summary, our work offers insights into the processing of modulated echo amplitude by FM bats. We have introduced a virtual-reality approach with time-variant targets to assess sensitivity to echo-amplitude modulation independently of echo-delay modulation. We have shown that FM bats are well capable of detecting modulations of echo amplitude despite the limitations that arise from the use of very short echolocation signals. We suggest that amplitude-modulation detection with echolocation not only differs fundamentally from delay-modulation detection, but also from amplitude-modulation detection in passive listening, due to the transient nature of the carrier signal. We speculate that the mechanism to detect (particularly the fast) modulations does not rely on the nominal cue alone, the echo amplitude, but on spectral cues that occur when the frequency modulation of the echolocation call interacts with the amplitude modulation of the target. Although we do not yet know whether FM bats make use of echo-amplitude modulations when they encounter a moving target, we provide an important proof-of-principle demonstration that offers release from the supposed trade-off between temporal and spatial acuity for FM bats.

## Abbreviations

AM: Amplitude modulation
CF: Constant frequency
FM: Frequency modulating/frequency modulation
ICI: Inter-call interval
SAM: Sinusoidal amplitude modulation
2AFC: Two-alternative forced choice
